# Integrative Transcriptomics and Proteomics Analysis of a Cotton Mutant *yl1* with a Chlorophyll-Reduced Leaf

**DOI:** 10.3390/plants13131789

**Published:** 2024-06-28

**Authors:** Hejun Lu, Yuyang Xiao, Yuxin Liu, Jiachen Zhang, Yanyan Zhao

**Affiliations:** 1 Institute of Crop Science, Plant Precision Breeding Academy, College of Agriculture and Biotechnology, Zhejiang University, Hangzhou 310058, China; 2 Xianghu Laboratory, Hangzhou 311231, China; 3 Plant Genomics and Molecular Improvement of Colored Fiber Lab, College of Life Sciences and Medicine, Zhejiang Sci-Tech University, Hangzhou 310018, China; xiaoyuyang_zstu@163.com (Y.X.); liuyuxin_zstu@163.com (Y.L.); zhangjiachen_zstu@163.com (J.Z.)

**Keywords:** cotton, transcriptomics, proteomics, chlorophyll, photosynthesis

## Abstract

Leaf color mutants serve as ideal materials for studying photosynthesis, chlorophyll metabolism, and other physiological processes. Here, we identified a spontaneous yellow-leaf mutant (*yl1*) with chlorophyll-reduced leaves from *G. hirsutum* L. cv ZM24. Compare to wild type ZM24 with green leaves, *yl1* exhibited patchy yellow leaves and reduced chlorophyll content. To further explore the mechanisms of the patchy yellow phenotype of the mutant plant, the transcriptomics and proteomics profiles were conducted for the mutant and wild types. A total of 9247 differentially expressed genes (DEGs) and 1368 differentially accumulated proteins (DAPs) were identified. Following gene ontology (GO) annotation and KEGG enrichment, the DEGs/DAPs were found to be significantly involved in multiple important pathways, including the obsolete oxidation-reduction process, photosynthesis, light-harvesting, the microtubule-based process, cell redox homeostasis, and the carbohydrate metabolic process. In photosynthesis and the light-harvesting pathway, a total of 39 DAPs/DEGs were identified, including 9 genes in the PSI, 7 genes in the PS II, 9 genes in the light-harvesting chlorophyll protein complex (LHC), 10 genes in the PsbP family, and 4 genes in the cytochrome b6/f complex. To validate the reliability of the omics data, GhPPD1, a DAPs in the PsbP family, was knocked down in cotton using the TRV-based VIGS system, and it was observed that the *GhPPD1*-silenced plants exhibited patchy yellow color, accompanied by a significant decrease in chlorophyll content. In conclusion, this study integrated transcriptomic and proteomic approaches to gain a deeper understanding of the mechanisms underlying the chlorophyll-reduced leaf phenotype.

## 1. Introduction

Cotton, as a crucial cash crop and raw material for the textile industry, holds significant importance for national economies and people’s livelihoods [1,2]. Photosynthesis is the main determinant of crop yield, and it is of great significance to study the mechanism of high yield in cotton from the perspective of photosynthesis [3]. Leaf color mutants serve as ideal materials for studying photosynthesis, chlorophyll metabolism, and other physiological processes [4]. The color of leaves is intricately connected to their photosynthetic assimilation capacity, which directly impacts the production and accumulation of organic matter in plants. During the growth and development of plant leaves, chlorophyll undergoes continuous synthesis and degradation, leading to the formation of a dynamic balance [5,6]. Disruption of this balance can result in leaf color mutations. Common plant leaf color mutations include yellow-green, albino, and light green color, as well as the appearance of stripes, and spots, each of which is often accompanied by abnormal chlorophyll metabolism and impaired chloroplast development [7,8,9]. These factors, in turn, affect the photosynthetic rate and yield of the plant.

The chloroplast vesicle membrane mainly contains four essential protein complexes, each playing a crucial role in the process of photosynthesis [10,11]. These complexes, namely photosystem II (PSII), cytochrome b6f complex, photosystem I (PSI), and ATP synthase, work in harmony to capture and convert light energy into chemical energy, enabling the synthesis of organic compounds essential for plant growth and survival [12,13,14]. PSII initiates the light-capturing process, while the cytochrome b6f complex facilitates electron transfer. PSI further harnesses light energy, and finally, ATP synthase generates ATP, the energy currency used by plants for various metabolic activities [15,16]. PSI and PSII are composed of their respective core complexes, which are responsible for the primary electron transfer events, as well as peripheral light-harvesting (antennae) complexes (LHC) [17,18]. The LHCs play a crucial role in capturing light energy and transferring it to the core complexes, where the process of electron transfer and energy conversion takes place. In plants, PSI-LHCI complexes exhibit a remarkable level of stability and uniformity [19]. The structure of the PSI-LHCI complex is composed of 12 core subunits, consisting of 9 membrane proteins (PsaA, PsaB, and PsaF-PsaL) and 3 water-soluble proteins (PsaC-PsaE) [20]. The key component of the PSII complex is the oxygen-excreting complex (OEC), which has been revealed to be responsible for the production of molecular oxygen during photosynthesis in *Porphyridium purpureum* [21]. In higher plants, the OEC consists of three extrinsic proteins, named PsbO (OEC33), PsbP (OEC23), and PsbQ (OEC16), respectively, which are essential for the proper functioning and stability of PSII [22,23].

In recent years, researchers have extensively investigated the molecular mechanisms underlying leaf color variation, and some genes associated with leaf color development have been identified [24,25,26]. While certain leaf color mutants have been discovered in cotton, the precise mechanism underlying leaf color changes in cotton remains unclear and requires further investigation. Hence, the identification of leaf color mutants in cotton, along with the exploration of regulatory genes, hold significant importance in unraveling the mechanisms underlying leaf color mutations. In this study, we identified a chlorophyll-reduced mutant *yl1* from the mutagenesis of *G. hirsutum* ZM24 and performed comparative transcriptome and proteome analysis on the mutants and ZM24 leaves to elucidate the molecular mechanism of leaf yellowing in cotton. Additionally, our study aimed to identify promising candidate genes that can be utilized for breeding cotton varieties with improved photosynthetic efficiency.

## 2. Results

### 2.1. Characterization of the Chlorophyll-Reduced Mutant yl1

**Figure 1:**
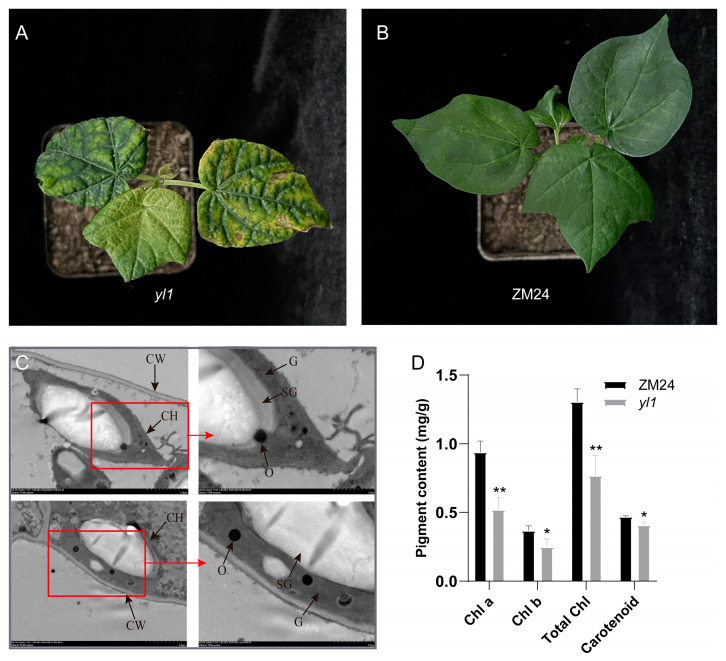
Plant phenotypes and pigment content (chlorophyll and carotenoid) of ZM24 and *yl1*. (**A**) Phenotype of *yl1* at three-leaf stage; (**B**) phenotype of ZM24 at three-leaf stage; (**C**) ultrastructure of the chloroplast of the wild-type and *yl1* leaves though TEM, CH, chloroplast; CW, cell wall; O, osmiophilic globule; G, grana lamella; SG, starch granule; (**D**) pigment (chlorophyll and carotenoid) content in young leaves of ZM24 and *yl1*. Leaves were picked from three plants of ZM24 and *yl1* for pigment content quantification, respectively. The asterisks indicate statistically significant differences, as determined by a Student’s two-tailed *t*-test (* *p* < 0.05, ** *p* < 0.01).

The chlorophyll-reduced leaf mutant was isolated from the spontaneous mutation of *G. hirsutum* ZM24, which exhibited patchy yellow leaf symptoms (Figure 1A,B), and designated as the *yl1* mutant. To estimate whether the difference in chlorophyll contents is caused by changes in chloroplast structure in mutants, we examined the ultrastructure of the chloroplast of the wild-type and *yl1* leaves using TEM (transmission electron microscopy). The ultrastructure analysis showed no significant difference in the starch granule, osmiophilic globule, or grana lamella between the wild-type and mutant chloroplasts (Figure 1C). The contents of chlorophyll (Chl) and carotenoid were measured, and the results showed that in *yl1*, the chlorophyll a, chlorophyll b, and total chlorophyll content were reduced by 44.5%, 32.9%, and 41.2%, respectively, compared to those of the wild-type (ZM24) plants (Figure 1D), indicating that the abnormal chlorophyll content in *yl1* may contribute to the patchy yellow leaf phenotype.

### 2.2. Differentially Expressed Gene Analysis between yl1 and ZM24

**Figure 2:**
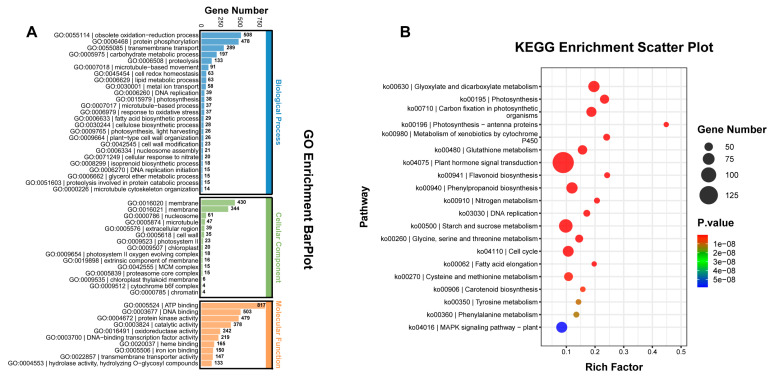
Gene ontology (GO) annotation and KEGG pathway enrichment of DEGs (q-value (FDR) of ≤0.01) between ZM24 and *yl1*. (**A**) Gene ontology (GO) annotation of DEGs between ZM24 and *yl1*; (**B**) KEGG pathway enrichment of DEGs between ZM24 and *yl1*.

In order to gain comprehensive insights into the genetic differences between the *yl1* and ZM24, we conducted RNA-seq analysis to capture the complete transcriptomes of their respective leaves. The identification of differentially expressed genes (DEGs) was determined by an adjusted q-value (FDR) of ≤0.01 and an absolute value of fold change of ≥2. According to these criteria, a total of 9247 DEGs were identified between ZM24 and *yl1*, including 5247 downregulated genes and 4000 upregulated genes. To analyze the biological functions of the DEGs, GO annotation and KEGG pathway enrichment analyses were performed. As presented in Figure 2A, the GO terms in the biological process category of most DEGs were primarily involved in the obsolete oxidation-reduction process, protein phosphorylation, transmembrane transport, the carbohydrate metabolic process, and proteolysis. It was noteworthy that a number of DEGs were involved in photosynthesis. In the cellular component category, a large number of DEGs were involved in the membrane, nucleosome, microtubule, extracellular region, cell wall, and photosystem II. Subsequently, the KEGG pathway enrichment analysis revealed that the DEGs were primarily associated with glyoxylate and dicarboxylate metabolism, photosynthesis, carbon fixation in the photosynthetic organisms, photosynthesis–antenna proteins, the metabolism of xenobiotics by cytochrome P450, and glutathione metabolism (Figure 2B).

### 2.3. Differentially Accumulated Protein Analysis between yl1 and ZM24

**Figure 3:**
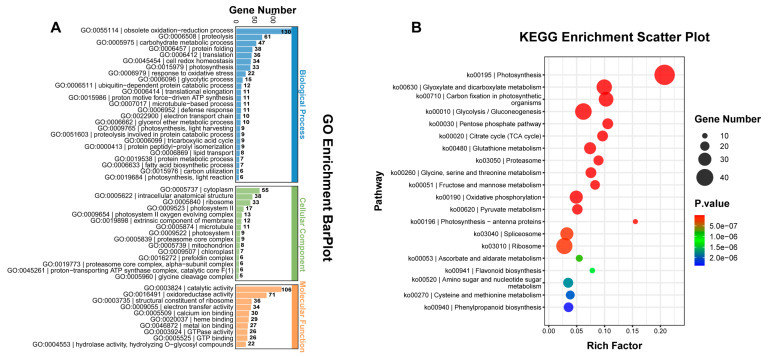
Gene ontology (GO) annotation and KEGG pathway enrichment of DAPs between ZM24 and *yl1*. (**A**) Gene ontology (GO) annotation of DAPs between ZM24 and *yl1*; (**B**) KEGG pathway enrichment of DAPs between ZM24 and *yl1*.

Proteome sequencing for the leaves of ZM24 and *yl1* was performed using the label-free technique. We identified a total of 2030 proteins, of which 1368 were classified as differentially accumulated proteins (DAPs), with 747 upregulated and 621 downregulated between the *yl1* mutant and ZM24 leaves. GO enrichment analysis was then used to annotate the DAPs as biological processes, cellular components, and molecular functions, respectively (Figure 3A). The major biological function categories included the obsolete oxidation-reduction process, proteolysis, the carbohydrate metabolic process, protein folding, translation, cell redox homeostasis, photosynthesis, and response to oxidative stress. In the cell component category, many DAPs were involved in the cytoplasm, the intracellular anatomical structure, photosystem II, the photosystem II oxygen evolving complex, and the extrinsic component of the membrane. In the molecular function category, the GO terms for the DAPs were predominantly involved in catalytic activity, oxidoreductase activity, the structural constituent of the ribosome, and electron transfer activity (Figure 3A). Further, the KEGG pathway enrichment analysis results revealed that the DAPs were primarily associated with protein processing in photosynthesis, glyoxylate and dicarboxylate metabolism, carbon fixation in the photosynthetic organisms, glycolysis/gluconeogenesis, the pentose phosphate pathway, and the citrate cycle (Figure 3B).

### 2.4. Integrated Analysis of Transcriptome and Proteome Data

**Figure 4:**
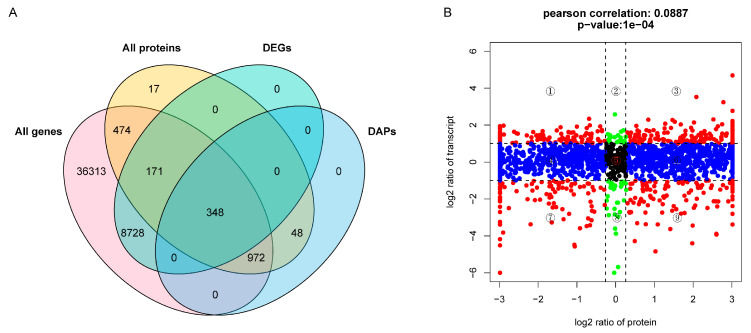
DEGs and DAPs association analysis. (**A**) Venn diagram of gene quantified in the transcriptome and proteome, DEGs, and DAPs. (**B**) Nine-quadrant map of gene and protein associations. Each dot represents a gene or protein; the values corresponding to each point in the horizontal and vertical coordinates indicate mRNA and protein expression differences, respectively.

**Figure 5:**
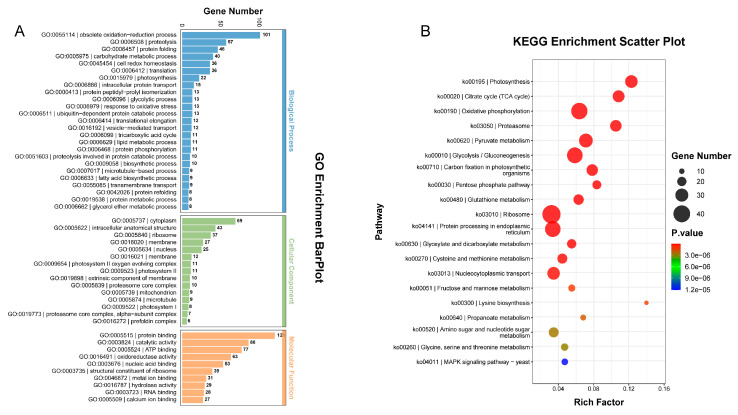
GO and KEGG cluster analysis of DAPs that showed differential expression at the protein level, but not the mRNA level. (**A**) Gene ontology (GO) annotation of DAPs; (**B**) KEGG pathway enrichment of DAPs.

To show the correlations between genes from the transcriptome and proteins from the proteome, a scatter plot was created in which each plot represented the log2 fold change of the ratio of protein: mRNA. In this study, a total of 1965 proteins/genes with quantitative information were divided into nine quadrants (Figure 4A,B). The 124 genes in quadrant 5 were not significantly different, as expressed at both the mRNA and protein levels. The 25 and 34 genes in quadrants 2 and 8 showed differential expression at the mRNA level, but not at the protein level. The 614 and 697 genes in quadrants 4 and 6 showed no differential expression at the mRNA level, but were differentially expressed at the protein level. The 104 and 114 genes in quadrants 1 and 9 showed a negative correlation at the protein and mRNA levels. The 161 and 92 genes in quadrants 3 and 7 showed positive correlation, and were both differentially expressed at the mRNA level and protein level (Figure 4B). Due to the fact that most DAPs were concentrated in quadrants 4 and 6, GO annotation and KEGG pathway enrichment analysis were performed for the DAPs in these two quadrants. GO analysis showed that the largest subcategories in the biological process were the obsolete oxidation-reduction process, proteolysis, protein folding, the carbohydrate metabolic process, cell redox homeostasis, translation, and photosynthesis. Notably, in the cellular component, certain DAPs were involved in the photosystem II oxygen evolving complex, photosystem II, and photosystem I (Figure 5A). Further, the KEGG pathway enrichment analysis results revealed that the DAPs were primarily associated with protein processing in photosynthesis, the citrate cycle (TCA cycle), oxidative phosphorylation, and carbon fixation in the photosynthetic organisms (Figure 5B).

### 2.5. Analysis of DEGs and DAPs Involved in Photosynthesis and Light Harvesting

**Figure 6:**
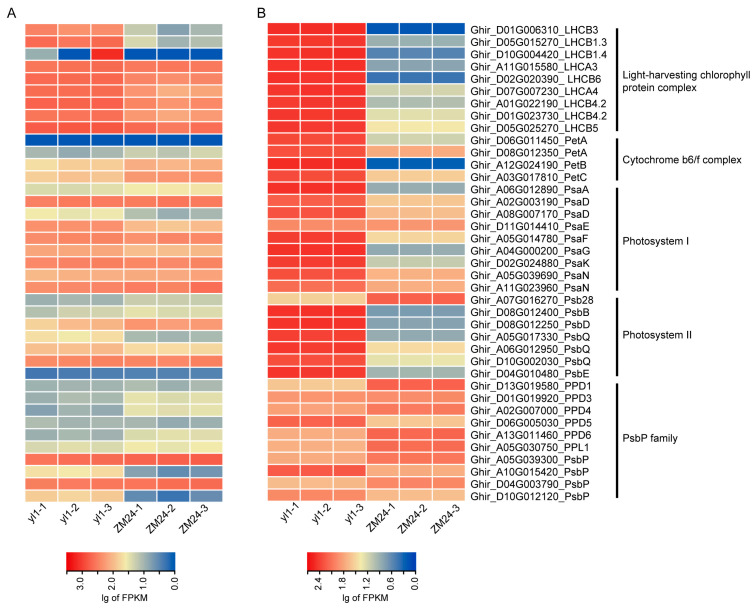
Heat map of the differential expression of DEGs and DAPs related to photosynthesis and light harvesting. (**A**) Differential expression of the genes between *yl1* and ZM24 in regards to mRNA level; (**B**) differential expression of the genes between *yl1* and ZM24 in regards to protein level.

It was noted that the GO terms related to photosynthesis and light harvesting (GO:0009765, GO:0015979) were enriched in both the DEGs and DAPs. Further investigation into the specific genes and proteins involved in these enriched GO terms could provide valuable insights into the mechanisms underlying the observed leaf color mutation of *yl1*. A total of 39 DAPs/DEGs were identified in these two GO terms, including 9 genes in PSI, 7 genes in PS II, 9 genes in the light-harvesting chlorophyll protein complex (LHC), 10 genes in the PsbP family, and 4 genes in cytochrome b6/f complex (Figure 6A,B). We randomly selected one gene from each type of photosynthesis gene for qPCR testing to verify the differential expression patterns (Figure S1). Among these genes, nine light-harvesting chlorophyll proteins and four cytochrome b6/f complex proteins were upregulated in *yl1* compared with ZM24. Two members in the PsbP family (Psb28 and PPD6) were downregulated in *yl1* compared with ZM24 in regards to both mRNA and protein levels. Four members of these two GO terms were found to be downregulated in *yl1* in regards to mRNA, but not protein, levels, including PetC, PsbD, PPD3, and PPD4. Four genes were found to be downregulated in *yl1* in regards to protein abundance, but not mRNA, including PPD1, PPL1, PsbP, and two PsbP genes (Figure 6A,B). These members may therefore be associated with chlorophyll deficiency.

### 2.6. Silencing of GhPPD1 Can Induce Leaf Yellowing

**Figure 7:**
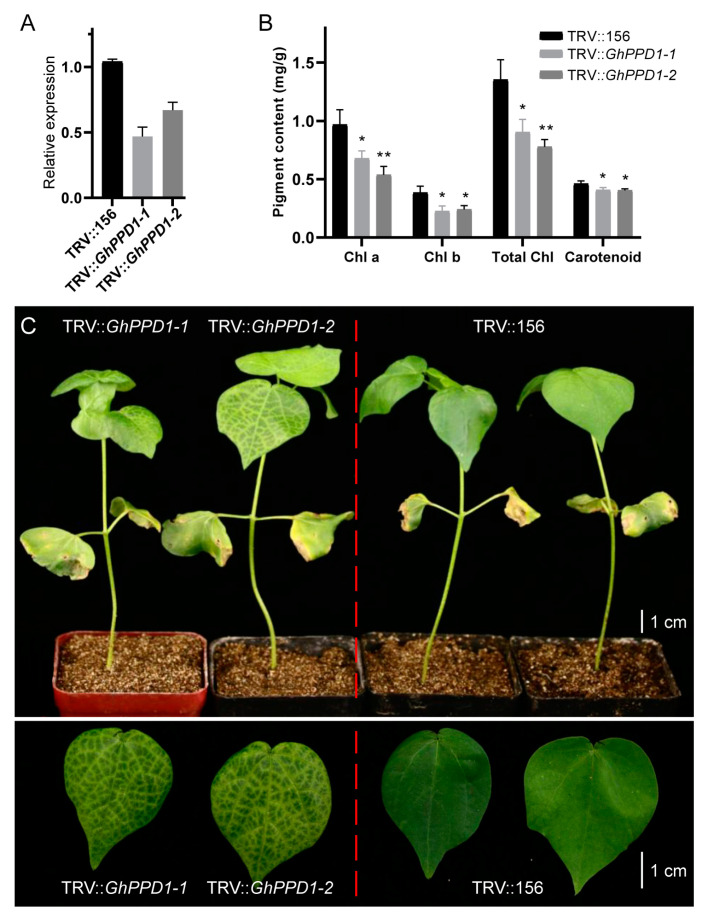
Effect of silencing of *GhPPD1* in cotton. (**A**) Relative expression level of *GhPPD1* in the leaves of *GhPPD1*-silenced (TRV::*GhPPD1*) and control (TRV::156) cotton; (**B**) pigment (chlorophyll and carotenoid) content in young leaves of *GhPPD1*-silenced cotton and control cotton; (**C**) phenotypes of whole plants (**top**) and the corresponding leaves (**bottom**) of *GhPPD1*-silenced and control cotton. For the qPCR and pigment content analysis, three biological repeats from each treatment were tested. The asterisks indicate statistically significant differences, as determined by a Student’s two-tailed *t*-test (* *p* < 0.05, ** *p* < 0.01).

To elucidate the role of *GhPPD1* in leaf color changes, a TRV-based VIGS system was used to reduce the expression of *GhPPD1* in cotton. The expression level of *GhPPD1* in *GhPPD1*-silenced plants (TRV::*GhPPD1*) was significantly decreased after VIGS silencing treatment compared to the level in the control (TRV::156). When the plants grew two to three true leaves, the color of the leaves was observed (Figure 7). The results showed that the leaves of the *GhPPD1*-silenced plants exhibited a patchy yellow color. Additionally, the contents of chlorophyll and carotenoid were detected, and the results showed that the chlorophyll a, chlorophyll b, and carotenoid contents were all significantly reduced in the *GhPPD1*-silenced plant compared to the ZM24. In conclusion, *GhPPD1* plays an important role in plant leaf color development.

## 3. Discussion

Fiber is the main yield trait of cotton. Cotton fiber is primarily composed of cellulose, which is a polysaccharide [27]. The synthesis and accumulation of cellulose occur through the carbon fixation and conversion processes in photosynthesis, playing a crucial role in the formation and development of cotton fibers [28]. The leaf color mutant is an important material for studying chlorophyll metabolism, chloroplast development, and photosynthesis. In recent years, leaf color mutants have been utilized to identify genes associated with leaf yellowing in various higher plants [29,30,31]. However, the understanding of the mechanisms underlying leaf yellowing development remains limited. In multiple plant species, the mechanism of leaf yellowing has been revealed through transcriptomic and proteomic analysis [32,33]. Currently, there is relatively limited research regarding the mechanism of leaf yellowing in cotton, and there have been few studies integrating transcriptomics and proteomics to reveal the regulatory mechanism of leaf color development in cotton. This study employs combined transcriptomic and proteomic approaches to better understand the critical regulatory mechanism of leaf color mutation in cotton. In plants, the process of photosynthesis primarily takes place within the chloroplasts and involves a series of reactions facilitated by two distinct photosystems, namely photosystem I (PSI) and photosystem II (PSII) [13]. GO terms associated with photosynthesis and light harvesting (GO:0009765, GO:0015979) were found to be significantly enriched in both the DEGs and DAPs (Figure 2A and Figure 3A), indicating that photosynthesis-related pathways play a crucial regulatory role in the leaf color mutation process. Conducting a comprehensive investigation of these enriched genes could yield valuable insights into the mechanisms underlying leaf color mutation [34]. Previous studies have indicated that leaf color mutants exhibit a downregulated expression pattern in regards to PSI proteins, PSII proteins, and LHC proteins [32,33,34]. For example, in paper mulberry, the iTRAQ-based quantitative proteomics analysis revealed that 26 DAPs encoding core proteins of photosystem I (PSI), photosystem II (PSII), and the light-harvesting chlorophyll protein complex (LHC) were suppressed in hybrid paper mulberry with golden-yellow leaves [34]. In contrast, in our study, 8 DAPs in PSI, 6 DAPs in PS II, and 9 DAPs in the light-harvesting chlorophyll protein complex (LHC) were upregulated in *yl1* compared with ZM24 in regards to the protein levels (Figure 6B). In fact, the phenotype exists in other previously reported mutants. For example, in pepper, KEGG enrichment analysis showed that under normal light conditions, the expression levels of 21 genes in LHC I (4 DEGs) and LCH II (17 DEGs) in the leaf-yellowing mutant R24 were significantly higher than those in WT [29]. It is possible that there are unique mechanisms for leaf yellowing in the pepper mutant R24 and the cotton mutant *yl1*, which requires further exploration. 

The PsbP family proteins exhibit unique functions in plants and are required for maintaining normal thylakoid architecture [23]. Currently, PsbP proteins have been categorized into three main groups: PsbP proteins, PsbP-like proteins (PPL), and PsbP domain proteins (PPD). In this study, ten DEGs/DAPs in the PsbP family were identified between *yl1* and ZM24, including four PsbP genes, five PPD genes, and one PsbP-like gene (Figure 6A,B). In Arabidopsis, the ppd1 mutant cannot grow photoautotrophically and specifically lacks PSI activity [23,35]. The function of *GhPPD1* in cotton has yet to be validated. In this study, transcriptome and proteomic analysis showed that *GhPPD1* exhibited no differences in expression at the mRNA level, but were differentially expressed at the protein level, indicating that GhPPD1 might function at the protein level (Figure 6A,B). To validate the reliability of the transcriptomic and proteomic data, the functions of *GhPPD1* were further investigated using the TRV-based VIGS system to obtain *GhPPD1*-silenced cotton plants. It was observed that the *GhPPD1*-silenced plants exhibited patchy yellow color, accompanied by a significant decrease in chlorophyll content (Figure 7C), indicating the important role of *GhPPD1* in leaf color development.

Chlorophyll is the primary photosynthetic pigment found in photosynthetic organisms, and its synthesis involves several enzymes, such as glutamyl tRNA reductase, magnesium chelatase, uroporphyrinogen decarboxylase, and coproporphyrinogen III oxidase (CPO). Magnesium chelatase is composed of three subunits, including CHLH (Mg-chelatase subunit H), CHLI (Mg-chelatase subunit I), and CHLD (Mg-chelatase subunit D). The coordinated action of these subunits is crucial for the insertion of the magnesium ion into protoporphyrin IX to form magnesium protoporphyrin IX. The mutations of the gene codes for the critical CHLH subunit can cause visible yellow phenotypes in Arabidopsis [36]. CHLM encodes a methyltransferase enzyme that catalyzes the methylation of magnesium protoporphyrin IX, an intermediate in the chlorophyll synthesis pathways. Mutations in the CHLM disrupt the methyltransferase process, leading to defects in chlorophyll biosynthesis [37]. The mutations or alterations of the genes encoding the enzymes involved in chlorophyll biosynthesis can lead to reduced chlorophyll accumulation. 

**Figure 8:**
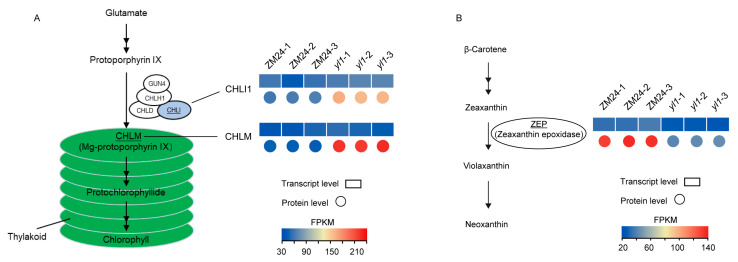
Protein expression changes occur in the processes of chlorophyll formation and carotenoid metabolism. (**A**) The proteins of CHLI1 (Ghir_A10G002740) and CHLM (Ghir_A07G005290) increased in the mutant leaves in comparison with green leaves; (**B**) Zeaxanthin epoxidase (ZEP, Ghir_A05G007800) was downregulated in protein level in mutant leaves compared with green leaves.

However, in this study, the protein levels of CHLI1 (Ghir_A10G002740) and CHLM (Ghir_A07G005290) were significantly increased in the mutant leaves (Figure 8); a similar research finding has been reported in the golden-yellow leaf of paper mulberry [34]. Carotenoids play a vital role in both photosynthesis and photoprotection. Given their ability to present a diverse range of colors, including yellow, orange, or red, carotenoids are considered one of the most important factors influencing the coloration of plant leaves [38]. Here, we found that the enzyme zeaxanthin epoxidase (ZEP, Ghir_A05G007800) was downregulated in the mutant leaves compared to the green leaves. The downregulation of ZEP will lead to the accumulation of zeaxanthin, a yellow carotenoid [38], in the leaf. A similar result was found in paper mulberry, where ZEP was suggested to play a crucial role in the formation of the golden-yellow leaf phenotype [34].

In this work, a PsbP protein was found to be downregulated in the mutant and was verified to induce a yellowing phenotype. The carotenoid metabolic pathways associated with leaf coloration have also been found to exhibit significant changes. The nonphotochemical quenching (NPQ) mechanism revealed the complex regulatory relationships in plant photosynthesis [3], which indicated that there were reasons for further investigating the photosynthetic pigment metabolism of green and mutant leaves.

## 4. Materials and Methods

### 4.1. Plants Materials

The yellow-green leaf mutant *yl1* was isolated from the spontaneous mutation of *G. hirsutum* L. cv ZM24. Plants were grown in a phytotron with a day/night temperature of 28/20 °C, and a light/dark cycle of 14/10 h. Leaves were collected from the same position (third-leaf samples), immediately frozen in liquid nitrogen, and stored at −80 °C.

### 4.2. Pigment Determination

Pigments were measured as described previously [34], with proper modifications. About 0.1 g of fresh leaves (avoiding leaves with thick veins) was weighed into a 2 mL centrifuge tube and ground into powder using liquid nitrogen, then 1.5 mL of 95% ethanol was added. The centrifuge tube was placed horizontally in a location protected from light until the leaves were completely white, then the tube was centrifuged at 12,000 rpm for 15 min, and the supernatant was removed. The supernatant was diluted the appropriate number of times (5×, 10×), and its absorbance values were measured at 665 nm, 649 nm, and 470 nm, respectively. Three biological replicates were performed for analysis; the raw data are provided in Tables S1 and S2.

### 4.3. Cytological Observation

The young leaves of three-leaves stage plants from the ZM24 and mutant *yl1* were used for TEM (transmission electron microscopy) observation. For the detailed method, see the work of Zhang et al. (2021) [39]. 

### 4.4. Liquid Chromatography–Tandem Mass Spectrometry (LC-MS/MS) and Database Search

LC–MS/MS analysis was conducted following a previously described protocol [40]. Mobile phases of gradient elution inlcuded a mixture of 0.1% formic acid and 2% acetonitrile in water (mobile phase A) and 0.1% formic acid in acetonitrile (mobile phase B). The peptides were solubilized in liquid chromatography mobile phase A and then separated using an ultra-high performance liquid chromatography system (nanoElute, Bruker Daltonics). The generated raw data from label-free LC–MS/MS were processed and searched against the *Gossypium hirsutum* protein database [41] using MaxQuant software (version 1.6.6.0; Cox Lab, Bavaria, Germany; https://www.maxquant.org/) for cotton protein identification and quantification. See Data Availability Statement for data accessibility.

### 4.5. Functional Analysis of Protein

To detect the differentially accumulated proteins (DAP), a one-fold change and *p* < 0.01 were considered. Heat maps were generated using TBtools [42]. For functional annotation and pathway enrichment, the DAPs were subjected to GO and KEGG pathway enrichment using OmicStudio tools (accessed on October 2023). GO and KEGG enrichment was performed based on Fisher’s exact test, with a significance cutoff set as *p* < 0.05.

### 4.6. RNA Sequencing and Data Analysis

The three young leaves were derived from three-leaf stage plants from the three independent biological replicates of ZM24 and *yl1*, respectively, for RNA-seq analysis. The RNA libraries were prepared and sequenced at Biomarker Technologies Corporation (Beijing, China). See Data Availability Statement for data accessibility. RNA-seq reads were then aligned to the reference TM-1 genome (HAU, version 1.0) using HISAT2 software [43]. The expression of each gene was quantified as fragments per kilobase of exon model per million fragments mapped (FPKM) using StringTie software v2.2.0 [44]. A one-fold change and corrected *p* value < 0.01 were set to detect the differentially expressed genes (DEGs). The DEGs were subjected to gene ontology (GO) and Kyoto Encyclopedia of Genes and Genomes (KEGG) pathway enrichment using the OmicStudio tools at https://www.omicstudio.cn/tool (accessed on October 2023).

### 4.7. Virus-Induced Gene Silencing of GhPPD1 in Gossypium hirsutum

The tobacco rattle virus (TRV)-based vector [45] was used to silence *GhPPD1* in *G. hirsutum*. In this study, a 212 bp fragment of GhPPD1-CDS was amplified from the cDNA of ZM24 using the primers TRV-PPD1-F and TRV-PPD1-R (Table S3) and inserted into the TRV binary vector pYL156 to construct a TRV::*GhPPD1* recombinant plasmid. The TRV binary vector pYL156 was used as a control plasmid and named TRV::*156*. The vectors were transformed into *A. tumefaciens* strain GV3101 and injected into fully expanded cotton cotyledons. Cotton leaves derived from 14 d seedlings were sampled for PCR, using the primers F and R to detect the transgenic plants. The interference efficiency of *GhPPD1* in transgenic plants was evaluated using quantitative real-time polymerase chain reaction (qRT-PCR), using the primers GhPPD1-F and GhPPD1-R (Table S1). Cotton Ubiquitin 7 (*GhUBQ7*, *Gh_D11G1120*) was used as the housekeeping gene. The 2^−ΔΔCt^ method was applied to calculate the relative gene expression levels; the qPCR result data are provided in Table S4.

## 5. Conclusions

In summary, this study employed a combination of transcriptomic and proteomic approaches between ZM24 and its chlorophyll-reduced mutant *yl1* to reveal the intricate mechanisms that contribute to the chlorophyll-reduced leaf phenotype. A total of 9247 DEGs and 1368 DAPs were identified. Some pathways that could potentially be linked to chlorophyll deficiency were revealed, including the obsolete oxidation-reduction process, photosynthesis, light harvesting, the microtubule-based process, cell redox homeostasis, and the carbohydrate metabolic process. The expression patterns of DEGs/DAPs involved in photosynthesis and light harvesting were further investigated to identify the functional genes. In summary, this study provided an important basis to further reveal the underlying mechanisms associated with the chlorophyll-reduced leaf phenotype in cotton. 

## Data Availability

The transcriptomes of *yl1* and ZM24 were deposited in NCBI with PRJNA1094870. The raw data for the proteomes were deposited in iProX (https://www.iprox.cn/page/home.html accessed on October 2023) with IPX0008953000.

## Supplementary Materials

The following supporting information can be downloaded at: https://www.mdpi.com/article/10.3390/plants13131789/s1, Table S1: Pigment content test data of ZM24 and yl-1; Table S2: Pigment content test data of control cotton, TRV::PPD1, and TRV::PPD2; Table S3: Primer sequences used in this study; Table S4: The qPCR raw data of *GhPPD1*-silenced sample and control; Tables S5 and S6: The qPCR raw data of five randomly selected DEGs; Figure S1: Five DEGs randomly selected for qPCR verification.
