# Supplementary material for: Integrative Transcriptomics and Proteomics Analysis of a Cotton Mutant yl1 with a Chlorophyll-Reduced Leaf
Source: Plants (Basel). 2024 Jun 28;13(13):1789. doi: 10.3390/plants13131789 (PMC11244299; doi:10.3390/plants13131789)
Supplement: Supplementary file 1 [file plants-13-01789-s001.zip › Figure S1.pdf]

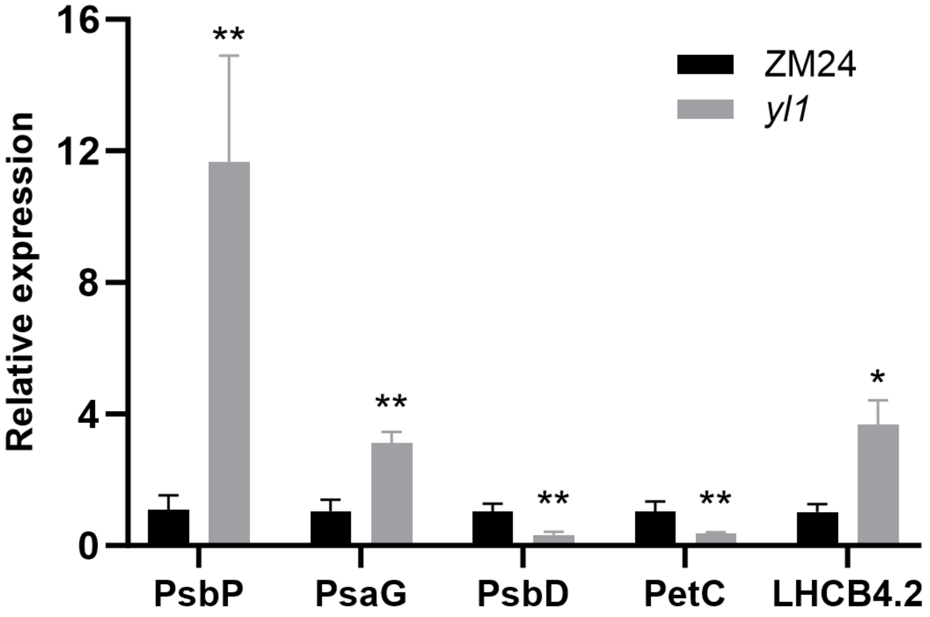

Figure S1. Five DEGs were random selected for qPCR verification. One gene from each type of photosynthesis genes (referenced to Figure 6A) were random selected and performed qPCR to obtain the relative gene expression difference between ZM24 and *yl1*. The $2^{-\Delta\Delta Ct}$ method was applied to calculate the relative gene expression level. Three biological replicates were performed for analysis. The asterisks indicate statistically significant differences as determined by Student's two-tailed t-test (*P < 0.05, **P < 0.01). The corresponding raw data see Table S5.
