# Supplementary material for: Integrative Transcriptomics and Proteomics Analysis of a Cotton Mutant yl1 with a Chlorophyll-Reduced Leaf
Source: Plants (Basel). 2024 Jun 28;13(13):1789. doi: 10.3390/plants13131789 (PMC11244299; doi:10.3390/plants13131789)
Supplement: Supplementary file 1 [file plants-13-01789-s001.zip › Supplemental files legend.pdf]

Table S1: Pigment content tests data of ZM24 and yl-1;

Table S2: Pigment content tests data of control cotton, TRV::PPD1 and TRV::PPD2;

Table S3: Primer sequences used in this study;

Table S4: The qPCR raw data of *GhPPD1*-silenced and control;

Table S5-S6: The qPCR raw data of five random selected DEGs;

Figure S1: Five DEGs were random selected for qPCR verification.
